# Human Endogenous Retroviruses in Multiple Sclerosis: Potential for Novel Neuro-Pharmacological Research

**DOI:** 10.2174/157015911795596568

**Published:** 2011-06

**Authors:** F.P Ryan

**Affiliations:** Department of Animal and Plant Sciences, Sheffield University, UK

**Keywords:** Multiple Sclerosis, autoimmunity, MS, human endogenous retroviruses, MS-associated retrovirus, MSRV, HERV-W, acute relapsing MS, secondary-progressive MS, human genome, genetic symbiogenesis.

## Abstract

There is growing evidence that the *env* genes of two or more human endogenous retroviruses (HERVs) of the W family are contributing to the inflammatory processes, and thus to the pathogenesis, of multiple sclerosis (MS). Increasing understanding of the human endogenous retroviral locus, ERVWE1, and the putative multiple sclerosis associated retrovirus, or MSRV, and in particular of the HERV-W *env* sequences associated with these, offers the potential of new lines of pharmacological research that might assist diagnosis, prognosis and therapy of multiple sclerosis.

## INTRODUCTION

Multiple sclerosis, or MS, has many of the features of an autoimmune disease, where the pathology involves an immune reaction to the body’s own cells or tissues – or to put it another way the failure of the adaptive immune system to recognise self. Useful, but inconclusive, information has come from the study of Mendelian genetics [1, 2], meanwhile, although the aetiology of MS remains uncertain, the innate and adaptive inflammatory processes involved are becoming increasingly understood at cellular and molecular level [3, 4]. There is evidence of genetic contribution to the autoimmune diseases. The human extended Major Histocompatibility Complex, or xMHC, is a key genetic region with regard to the evolution, and expression, of our adaptive immunity, with 22% of its 421 genes believed to have immunoregulatory function, and various genes associated with auto-immunity [5]. There is also growing evidence that human endogenous retroviruses, or HERVs, have played an important role in the evolution of the xMHC, and it is likely that they continue to play an important role in its function and malfunction [1, 6, 7]. In addition we now know that HERVs and related genetic sequences have contributed to the evolution and structure of the human genome *per se*, with implications for human embryology and physiology [8]. The logical first step in a systematic approach to understanding this complex situation is to examine the nature of HERVs and their importance in the evolution, genetic structure and function of the human genome.

## HUMAN ENDOGENOUS RETROVIRUSES

We are familiar with viruses as the causative agents of infectious diseases, but we also need to recognise that, from the evolutionary perspective, they are also capable of bringing about genetic change in the germ lines of their hosts. This is known as viral symbiogenesis and it has major evolutionary potential [9]. Conceptually and mechanistically, this differs from the more familiar concept of genetic change through mutation, although it is complementary rather than contradictory to the classical concepts of evolution through mutation and selection.

Symbiosis is defined as the living together of different organisms. The partners in a symbiotic relationship are known as “symbionts” and the partnership, regardless of the level of the symbiosis, is known as the “holobiont”. While it includes mutualism, symbiosis also includes parasitism, where one partner benefits to the detriment of another, and commensalism, where a partner benefits without detriment to the other partner, or partners. In fact mutualism very often derives from parasitism, and over time the nature of the relationship can involve dynamic change, which will also be reflected in how natural selection operates. When symbiosis gives rise to evolutionary change in one or more of the partners, it is known as symbiogenesis. Symbioses take place at various levels, depending on the precise nature of the interaction. In lichens, for example, it involves the sharing of metabolites between algae and fungi, so this is known as a metabolic symbiosis. Other symbioses, such as those of the cleaner stations on the ocean floor, involve the sharing of complex behavioural patterns between large fish and small cleaner fish and crustaceans. These are examples of mutualistic symbioses in which the partners bring pre-evolved abilities to the holobiontic partnership. In all such mutualistic partnerships, even where the symbionts still reproduce independently, selection must work to some degree at partnership level.

Viral symbiosis can also work at various levels, for example through culling of host species gene pool as part of a mechanism known as “aggressive symbiosis” [10]. This is probably a feature of most, if not all, viral epidemics, and it becomes particularly important when viruses enter into a persistent strategy with their hosts after initial infection [11]. Viral persistence, for example in the rabbit myxomatosis plague, or in the present human pandemic of AIDS, changes the virus-host evolutionary dynamic, making it more likely that selection will operate to a significant degree at partnership level. Moreover, with retroviruses, there is an additional, powerful evolutionary dynamic that has played an important role in the evolution of the human genome, and thus becomes an important consideration in the pathogenesis of disease. This is the process of endogenization, where the infecting retrovirus inserts its genome into the germline of the host. This pattern of “genetic symbiogenesis”, involving the fusion of the two genomes of former parasite and former host, gives rise to a novel holobiontic genome. Viruses behaving selfishly within a germ line would threaten survival. Thus selection, at this stage, must operate at the level of the holobiontic genome, selecting for sequences of former host or virus that enhance survival and selecting against former host or viral sequences that impair survival. This offers the potential for powerful, and relatively rapid, evolutionary change. To understand the contribution of viral symbiogenesis to the evolution of the human genome, we need to look more closely at the genetic structure of a retrovirus.

## THE GENOMIC ORGANISATION OF RETROVIRUSES

**Fig. (1):**
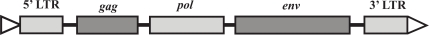
There are three genetic domains, flanked by the regulatory LTRs, or long terminal repeats. The *gag* domain codes for matrix and core shell proteins, the *pol* codes for reverse transcriptase, protease, ribonuclease and integrase, and the *env* codes for the surface and trans-membrane glycoproteins. The *env* of a HERV-W is also responsible for syncytiotrophoblast cell fusion in the human placenta –see Fig. (4).

The basic retroviral genome comprises three genetic domains, conventionally referred to as the genes, *env*, *gag* and *pol*, which code for a variety of proteins. (Fig. **1**) For example *gag *codes for the proteins necessary for viral assembly, including matrix and core shell proteins, *pol *codes for the enzymes necessary for viral replication, such as reverse transcriptase, protease, ribonuclease and integrase, while *env* codes for the surface and trans-membrane glycoproteins. In particular, the *env*-associated proteins are essential for the binding of virus to cell-surface membranes and they have been specifically associated with neurovirulence [12]. In virus-host interactions, these same proteins give rise to host cell fusion and immunosuppression. Thus the viral genome is a holistically functional unit whose genes, and flanking regulatory regions, known as LTRs, are pre-evolved to manipulate key aspects of human immunity, physiology and genetics. Invading retroviruses will often endogenize the germ lines of their hosts again and again, with up to a thousand or so different sites of integration randomly distributed throughout the chromosomes, each integration site offering the possibility of future symbiogenetic potential. In human terms, although many such insertions have been “switched off” by the policing action of selection, such has been the massive volume of viral insertions that a significant number have positively contributed to the virus-host union, adding novel genetic complexity to the evolving holobiontic genome.

The human genome contains between 30-50 different HERV families, subdivided into some 200 different subgroups, most of which resulted in multiple integrations, and most of which originate from exogenous retroviral invasion during the pre-human phase of our evolutionary history. The families and subgroups are commonly characterised on the basis of the single-letter code for the amino acid complementary to the t-RNA primer binding site that initiates transcription. Thus, for example, a HERV-K initiates transcription with lysine and HERV-W with tryptophan. Each family and subgroup is thought to represent an independent evolutionary lineage, which would imply that the ancestral human genome has been subject to a large series of independent retroviral colonisations. Although many of these unions took place during our mammalian ancestry, at least eight full length HERV-K proviruses are unique to humans having entered the human germ line after humans diverged from chimpanzees [13, 14].

The symbiogenetic potential of such massive viral colonisation of the human genome is likely to be major. However, if we further consider that viral lineages, in particular those where the viral genes and regulatory sequences are conserved within the flanking viral regulatory LTRs, retain much that is quintessentially viral in structure and behaviour, we have an additional potential for the genetic underpinning of disease.

**Fig. (2):**
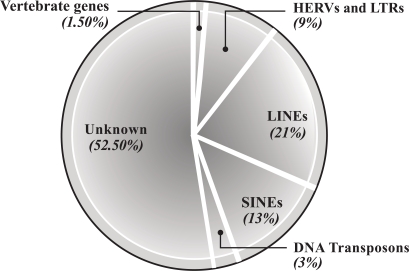
The “vertebrate” section contains the genes historically associated with coding for proteins, comprising exons separated by introns. The HERV section contains all of the HERV inserts, including complete HERVs, defective HERVs and isolated LTRs. The LINEs and SINES (long interspersed and short interspersed repetitive elements) are classed as retro-transposons. The DNA transposons probably originated through DNA viral insertion.

**Fig. (3):**
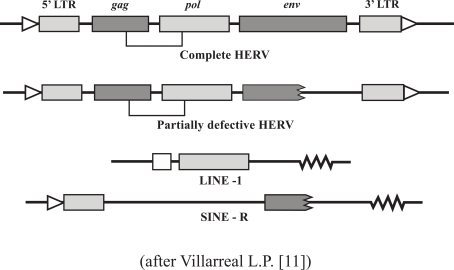
Incomplete HERVs are derived from mutations affecting the various genetic domains and LTRs. There are two conflicting theories for the origins of the LINEs and SINEs. One theory posits an independent evolution within the genome. The other posits that they are breakdown products of endogenous retroviruses, which subsequently developed their own evolutionary trajectories. Functionally they interact with one another, so should be considered as related groups.

These virus-related sequences amount to almost half the total human DNA. (Fig. **2**) HERVs and the retrotransposon elements known as LINEs and SINEs (Fig. **3**) have been incorporated into human embryology and day-to-day genetic chemistry and physiology [8, 15, 16]. Human endogenous retroviruses, and the retrotransposon elements, have also been discovered in the diseased cells and tissues of very many human diseases, and the majority of cancers and auto-immune diseases [1, 17]. Research into the role of HERVs and retrotransposon elements in human evolution is at an early stage, and understanding of the potential role of HERVs and retrotransposons in this wide spectrum of disease is hampered by ignorance of the role they are playing in human development and normal physiology. This inevitably extrapolates to our understanding of the role of HERVs in MS, although there is increasing evidence that the HERV-W family is playing a significant role in the pathogenesis. It is illuminating to examine the specific HERV-W locus known as ERVWE1.

## THE ERVWE1 GENETIC LOCUS

Syncytin-1 is a viral protein that plays an essential role in the differentiation of the syncytiotrophoblast cells of the human placenta [18]. The ERVWE1 locus, a HERV-W insertion that codes for syncytin-1, is located on human chromosome 7 (7q21.2). Its genetic structure is shown in Fig. (**4**), while a more detailed view of its regulatory region is shown in 4b [19]. It is clear that the locus contains a complete HERV-W, with *gag*, *pol* and *env* genes flanked by the regulatory LTRs. We also see that the viral genome is further flanked by the isolated LTRs of a second endogenous retrovirus of the MaLR family. The locus is referred to as an ERV rather than HERV because humans share the locus with the great apes. It derives from an exogenous retrovirus (the forbear of the HERV-Ws) that invaded the primate genome between 20 and 30 million years ago, and while the *gag* and *pol* genes have been inactivated by deletions and stop codons, the *env* gene has been conserved in humans, chimpanzees, gorillas and orang-utans. This *env *gene codes for the protein syncytin-1. It is promoted by the 5’HERV-W LTR, which has also been selectively conserved. An additional upstream regulatory component involves the conserved 5’ MaLR LTR. So we have a viral gene within the structural unit of a viral genome, regulated to important degree by two viral LTRs, which expresses a conserved viral protein that is essential to normal human reproduction.

ERVWE1 is far from unique in its viral contribution to human evolution and reproduction. We now know that there are at least seven additional endogenous retroviral elements that contribute to human reproduction, including HERV-FRD, ERV-3, HERV-E.PTN, LTR10A, HERV-H7/F(XA34), HERV-Fb1, and HERV-HML6-c14, [8, 10]. There is some evidence, albeit preliminary, that a number of different HERV families may be contributing as yet unknown roles in the physiological function or cyto-chemical structure of the normal human brain [6, 8], as well as early evidence that HERVs may contribute to diseases involving the central nervous system [1]. There are two additional considerations of holobiontic viral integration that need to be grasped. Where a specific HERV has inserted many times into different chromosomes, and where one or more of its insertions have become conserved for an essential genetic function, selection is likely to exert a “policing” control on the unwanted expression of closely homologous genetic sequences from other insertion sites, which might otherwise give rise to dysregulation of the conserved role. This is further complicated by the fact that viral sequences, even if truncated or silenced by stop codons, may be capable of expression and even the unstopping of stop codons, through recombination between related HERVs [1, 20], although such mechanisms have yet to be confirmed in humans. There is an intriguing, though as yet tentative, suggestion that recombination of normally silenced HERVs and sequences might play a role in disease [21, 22].

## THE MS-ASSOCIATED RETROVIRUS

In 1997, reporting for a collaboration of Swiss, French and British scientists, Perron described the isolation, and partial molecular identification, of novel retrovirus particles from cell cultures of the leptomeninges, choroid plexus, and peripheral blood B lymphocytes of MS patients. They also found related genetic sequences in the CSF and cell-free plasma of non-treated patients but not from matched controls [23]. They named the virus the MS-associated retrovirus, or MSRV. The authors were uncertain whether they were dealing with an exogenous, infectious retrovirus or a replication competent, virion-producing HERV. Subsequently MSRV genetic sequences were found to share a close homology with the HERV-W endogenous family, and MSRV was subsequently regarded as the founder discovery of this endogenous virus family [24]. Continuing uncertainty as to whether the virus was exogenous or endogenous led to the MS-associated virus being relabelled the MSRV/HERV-W, but for convenience the virus will be referred to as MSRV in this text. In 2003 Nowak and Polish colleagues found MSRV *pol* sequences in the serum and peripheral blood lymphocytes of patients with other neurological conditions as well as controls [25]. These sequences appeared to be endogenous and widely dispersed through the chromosomes. Perron also confirmed that *gag* and *env* proteins encoded by the MSRV are expressed by normal cells in the central nervous system [26]. However, the expression of such proteins in neurones of normal subjects was modest in density and compatible with some physiological function. Meanwhile there was an accumulation of *gag-*encoded protein in neurones within demyelinated white matter in MS-affected brains, and prominent *gag*-encoded protein was found in endothelial cells within MS lesions from acute or actively demyelinating cases, a pattern that was not seen in controls.

In Sardinian patients – Sardinia appears to suffer a relatively high incidence of MS – the Italian researchers, Dolei *et al.*, confirmed the detection of MSRV in the plasma of 12.8% of healthy blood donors, meanwhile they found the virus in the plasma of all 39 MS patients tested, which included 24 suffering a relapsing remitting course, 4 secondary-progressive and the remainder newly diagnosed [27, 28]. The differences between MS and control findings was highly significant (p > 0.000001). They also detected the virus in the CSF of 80.6% of MS patients compared to 40% in a small cohort of controls with other neurological diseases, such as CNS vasculitis, trigeminal neuralgia, ALS and recurrent optic neuritis of unknown origins. Further analysis of the latter groups suggested that MRSV presence in blood tallied with the inflammatory nature of the diseases. On first presentation all MS patients had detectable virus in their plasma, and 50% had detectable virus in the CSF. MSRV *env* and *pol* RNA transcripts were also detected, albeit at low levels in normal human blood and brain, but these showed a 20- to 25-fold increase in brain samples from MS patients. Increasing CSF titres of the virus over time correlated with both duration of disease and disease severity as ranked by standard clinical diagnostic criteria. They also showed strong immunoreactivity for MSRV in specific MS lesions in brain when compared to controls, with intense staining for MSRV *env *expression in chronically active MS lesions, where it was localised to microglia and astrocytes. A similarly intense staining for *env* expression was found within astrocytes at the plaque core.

This work confirmed a possible association between MS and expression of the putative MSRV, but uncertainty continued as to whether the virus was playing a role in pathogenesis, as opposed to a physiological reaction to the disease, or perhaps no more than an epiphenomenon associated with inflammatory processes *per se*.

## MSRV/HERV-W – PATHOGENETIC OR PHYSIOLOGICAL?

Retroviral *env* proteins are known to give rise to neuro-toxicity through a variety of direct and indirect mechanisms, for example through inducing glial cells to produce neuro-toxic chemicals. The latter can implicate cells involved with innate immunity, such as macrophages, microglia and astrocytes, or cells involved with adaptive immunity, such as T or B lymphocytes, all of which have been variously linked to the pathology of MS [4, 12]. Retroviral neuro-toxicity can also involve complex interactions between exogenous viruses and endogenous retroviruses and host genetic variations that control the expression of the latter [29]. In 1997, Ménard, reporting on behalf of a multi-centre group of French and UK colleagues, found significant correlation between glial cell toxicity and reverse transcriptase activity in monocyte/macrophage culture supernatants in MS patients when compared to controls [30]. In 2001 Perron and colleagues showed that inoculation of peripheral blood lymphocytes (PBLs) of healthy controls with MSRV virion particles led to a significant polyclonal activation of T cells following the pathogenetic pattern of a superantigen [31]. A hybrid animal model grafted with human lymphocytes and injected intraperitoneally with MSRV led to fatal brain haemorrhages, with evidence of systemic viraemia and cytokine activation in the MSRV-injected animals [32], a response compatible with a T-cell mediated immunopathogenicity [33]. In 2002, the Italian group examined a further 15 MS patients, who had not been treated with immunomodulatory drugs for at least 3 months before taking CSF samples [34]. Nine of the 15 had demonstrable MSRV in their CSF. While the EDSS disability score and MRI findings showed no significant difference between patients with or without the virus at onset, the virus-positive cohort suffered more relapses and succumbed to a more disabling course over three years of follow-up. The authors concluded that the presence of MSRV in CSF at clinical onset of the disease correlated with disability progression. A positive feedback loop affecting MSRV expression was subsequently reported by the same group, where viral release from peripheral blood mononuclear cells was stimulated by the cytokines interferon-γ and TNFα, both known to play a detrimental role in MS, whereas viral release was inhibited by interferon-β, a therapy of proven efficacy in MS [35].

In 2004, Antony presented the findings of an international collaboration that included Canadian, US, French and UK scientists, demonstrating increased expression of HERV-W-*env* encoded protein in the brains of MS patients as opposed to normal controls [36]. Western blot testing also showed that syncytin-1, the protein encoded by the ERVWE1 locus, showed a three-fold increase in expression in brain tissue from MS patients as compared to controls. Syncytin-1 immuno-reactivity was also found in activated glial cells within acute MS lesions and these cells also showed inducible nitric oxide synthase (iNOS) immunoreactivity, which suggested ongoing inflammation. Because astrocytes and microglia are important modulators of neuroinflammation, they looked further at syncytin-1 regulation within these cells and confirmed that it was selectively upregulated in astrocytes and microglia from individuals with MS, but not in other neural cells, including neurones and oligodendrocytes. These findings showed similarities with what had been demonstrated by the Italian researchers testing for MSRV. However, based on the HERV-W *env *sequences, as compared to other HERV-*env *sequences in GenBank, the authors concluded that the *env* expressed in their experiments was syncytin-1 coded by the *env *gene of the ERVWE1 locus. Constructing a vector that would efficiently express the same HERV-W-*env* sequence within cells, they infected cell cultures of foetal astrocytes and macrophages with the vector and looked for syncytin-1-mediated induction of neuro-inflammatory genes and their products. Within 24 hours of vector infection, the proinflammatory cytokine IL-1β was significantly increased in both cell types compared to controls. Inducible iNOS, another potent neurotoxin, was also increased in astrocytes but not in monocytes, meanwhile the anti-inflammatory cytokine IL-10, was not induced by the vector. This suggested that syncytin-1 expression in astrocytes and macrophages selectively induced proinflammatory responses.

Oligodendrocytes are the cells that produce myelin, and are the principal cell associated with the demyelination involved in the pathogenesis of MS. When human oligodendrocytes were exposed to the conditioned medium from cultures of human foetal astrocytes and monocytes, the medium from the syncytin-1-stimulated cultures proved to be highly toxic to the oligodendrocytes when compared to the medium from non-syncytin-1-stimulated controls. This toxicity included a range of observed effects from retraction of cellular process to cellular death. On the basis that the toxicity was in part the result of redox reactants, such as nitric oxide, they looked at the potential benefit of scavenging redox reactants, using the polyphenolic antioxidant, ferulic acid, a non-steroidal anti-inflammatory-based-antioxidant. When introduced into the culture medium, this markedly reduced the lethality to the oligodendrocytes. The authors went on to demonstrate a similar neuro-toxicity related to implantation of the syncytin-1 vector into the brains of mice, confirming uptake and syncytin-1 expression in astrocytes, followed by a decrease in oligodendrocyte numbers, accompanied by neuro-behavioural dysfunction on performance testing. The latter also appeared to be improved by concomitant treatment with ferulic acid.

By now the weight of evidence, from study of both MSRV and the ERVWE1 locus, suggested that two HERV-Ws, and in particular their *env *genes, were likely to be implicated in the neuroinflammation of MS, and perhaps even the pathogenesis. In 2007 Antony and colleagues attempted a comparative study of the pathogenetic effects of ERVWE1 and MSRV [37]. The two viruses were known to be members of the same HERV-W family, with the *pol *sequences sharing 92% of their genetic identity, making it difficult to differentiate between the viruses using conventional probes. But the *env* sequences of ERVWE1 and MSRV had been reported by Mallet to share 81% identity [18], which might make differentiation easier. For example research into the genetic coding for the surface unit of the HERV-W envelope protein, a 293 amino acid fraction, led to the recognition of a variety of genetic “clones”, each coding for a different variant of ENV-SU, with possible differences in their pathogenetic potential. The clone described by Mallet as representative of MSRV *env* was based on the GenBank accession number AF331500. However, Antony and colleagues based their MSRV *env* sequences on another clone, with the Gen-Bank accession number AF123882.

When they compared post-mortem brain tissues from patients with MS to controls, they found significant increases in the detection, and expression, of ERVWE1 *env* in MS brains while finding no increase in detection or expression of the AF123882 clone. They also found marked differences in the expression of *env*-encoding RNA in plasma and CSF samples of both patients and controls between the two different *env* sequences. Where ERVWE1 *env *was found in 84% and 62% of plasma samples from MS versus non-MS patients and from 64% and 75% of CSF samples from MS versus non-MS patients. Meanwhile the AF123882 clone was detected in just a few plasma samples and in none of the CSF samples of both MS patients and controls. The high incidence of ERVWE1 expression in normal controls is not surprising since research, as yet unpublished, by Larsson and colleagues in Sweden has shown dense expression of syncytin-1 in normal brains [personal communication]. Although the function of syncytin-1 in normal brains is currently unknown, its regulation is complex and this might allow for a distinct physiological role in the normal physiology of astrocytes, other glia, neurones or other cells or tissues within the central nervous system. Antony’s results contrast with the findings of the Italian group [30-35], but the MSRV *env* sequence employed by the two groups appears to differ. Laufer *et al.,* would subsequently show that the AF123882 MSRV *env* clone, taken as representative of MSRV in the Antony study, represented an incomplete *env *open reading frame [22]. Two years later, when Rolland *et al.* looked at the clone AF331500, which they regarded as the ENV-SU of the MSRV – the envelope fraction that interacts with host cells – they found that it induced human monocytes to produce major inflammatory cytokines through engagement of CD14 and TLR4, which are pattern recognition receptors of primary importance in innate immunity [38]. This same ENV-SU could also trigger a maturation process in human dendritic cells and endow dendritic cells with the capacity to support a Th1-like pattern of Th cell differentiation.

Overviewing the main published findings of the different groups, it seems that while both MSRV and ERVWE1 are associated with MS, the two are distinctly different members of the HERV-W family and they show differential expression and detection in MS patients’ tissues, cells and plasma. Both ERVWE1 *env* and MSRV *env* showed increased expression in the brains of MS patients when compared to controls. However, some caution is necessary in the interpretation of cellular detection, since the genetic sequences of the two viruses share such a close homology it is currently impossible to differentiate between them [26]. Clearly, the development of specific antibodies or primers capable of intracellular differentiation would be a major step towards understanding their differential contribution towards pathogenesis. An earlier study had suggested that raised levels of HERV-W and HERV-K in MS brains were secondary to activation by cytokines such as TNFα [39]. In 2007, a cooperative study involving the Italian researchers and colleagues in Philadelphia, examined the regulation of ERVWE1 in astrocyte cell cultures, showing that cytokines known to play significant roles in the inflammatory process of MS, such as TNFα, interferon-γ, interleukin-1 and interleukin-6, activated the ERVWE1 promoter, while interferon-β, the therapeutic drug of proven efficacy in acute relapsing pattern MS, was inhibitory [40]. In particular they showed that TNFα activation of the promoter worked through a subunit, known as p65, within the enhancer domain of the promoter. TNFα activation was epigenetically abolished by siRNA directed against p65 – indicating a possible therapeutic/ pharmacological target. They also excluded the possibility that increased expression of syncytin-1 in MS was caused by mutational change in the ERVWE1 promoter. This led them to assume that the increased expression of ERVWE1 was related to the inflammatory microenvironment, and specifically the activation of the promoter by proinflammatory cytokines. Highlighting the marked similarity of cellular location, expression and potential neuro-toxicity of both MSRV and ERVWE1 in MS patients throughout a range of different studies in different research units, and demonstrating that, in astrocytes, the ERVWE1 promoter is activated and inhibited by the same cytokines, and interferon-β, as shown earlier with MSRV, they concluded that both MSRV and ERVWE1 are likely to be activated *in vivo* in MS patients.

The growing link between a HERV-W *env* gene and MS was highlighted when the Italian group – who had earlier confirmed a link between detection of MSRV in CSF on first presentation with eventual poorer prognosis – now showed that the measurable viral load of MSRV in the blood and cerebrospinal fluid of MS patients correlated closely with clinical activity score, stage and progression of MS. Blood levels of the virus fell below detection limits in the majority of a group of patients after three months of beta-interferon therapy, suggesting that plasma titre of MSRV might be useful both in prognosis and response in patients undergoing medical therapy [41]. In 2007 further research by Antony *et al., *showed that syncytin-1 regulates neuroinflammation and its receptor expression in MS. In particular ASCT1, a receptor for syncytin-1 and a neutral amino acid transporter, was selectively suppressed in astrocytes in the brains of MS patients. This paper included important new research on cultured astrocytes and further evidence that epigenetic blocking of the action of syncytin-1-induced suppression of ASCT1, and thus the release of oligodendrocyte cytotoxins by astrocytes [42]. By now the weight of evidence was suggesting that both MSRV and ERVWE1, and specifically their *env *sequences, were contributing to the pathogenesis of MS. Moreover, studies in several centres were also indicating possible targets for pharmacological intervention [35, 36, 40-42].

In a further study, Mameli and colleagues searched for a reliable means of differentiating MSRV *env *from syncytin-1 sequences [43]. They examined twelve variants of MSRV *env* and eight variants of syncytin-1 sequences, comprising all those deemed suitable from GenBank as well as those detected experimentally in their own cohort under study. From this exhaustive group, they discovered a 12-nucleotide insertion in the trans-membrane moiety of the MSRV *env* gene, which was present in all twelve MSRV *env* variants they tested but was absent from all of the syncytin-1 variants. Based on this newly-discovered insertion, they developed discriminatory real time PCR assays that could selectively amplify either MRSV *env* or syncytin-1. Previous data had shown that both MSRV and ERVWE1 were expressed in the brains of MS patients, while only MSRV was found in peripheral blood plasma, and was expressed by cultures of peripheral blood monocytes (PBMCs) in blood-positive individuals. Also, while syncytin-1 had been found intracellularly and on the plasma membrane, it had not been detected extracellularly and its sequences were not expressed in the MSRV virus-like particles, which were visible on electron microscopy, and contained demonstrable reverse transcriptase activity and all three HERV-W genes. This appeared to fit with the fact that ERVWE1 was exclusively expressed at cellular level, as would be expected of endogenous sequences, meanwhile MSRV sequences were also expressed extracellularly, as would be expected of circulating virions. Now, using their newly constructed PCR assay, Mameli and colleagues were able to compare and contrast the expressions of HERV-W generic *env, *MRSV *env* and ERVWE1 *env *(syncytin-1) expression in the plasma, in cultured PBMCs and in the supernatant of the cultured cells, in four MS patients who had not yet been treated, in four MS patients who had been treated and in six healthy blood donors acting as controls. The results, albeit in small numbers of patients, were striking.

The controls showed no expression of any of the three *env* sequences. The untreated MS patients showed high levels of expression of MSRV *env *in all three test situations, with markedly reduced titre of expression in the treated MS patients. A similar, but much lower level of expression of generic HERV-W *env *sequences was seen in all of the MS patients. Meanwhile there was no measurable expression of ERVWE1 *env *(syncytin-1) in the plasma or culture supernatant of any of the MS patients, and it was only expressed in very low titre in a single untreated MS patient within the PBMCs. The authors concluded that these patterns of *env *expression confirmed the link between MS and MSRV.

## RE-EXAMINING THE BASICS

A consistent, and important, uncertainty revolves around the question whether the MSRV is an exogenous infectious virus or the expression of an endogenous virus, or endogenous sequences. The finding of extracellular virion particles does not necessarily imply an exogenous virus, since virion particles have been reported in the expression of HERVs, although these do not usually contain complete viral genomes. In a more general sense, the widespread discovery of HERV expression in autoimmune diseases and cancer, and the specific instance of HERV-Ws in MS, highlights the need for a fundamental methodological approach to HERVs in disease. In 2009, a study by Laufer and colleagues provided a role model for such a basic approach in examining a large number of transcribed loci of HERV-W *env *sequences within the human genome, and aiming to clarify the MS-related retrovirus *env* contribution [22]. Pavlíček and colleagues had already shown that the HERV-W family has inserted into roughly 650 positions dispersed throughout the human chromosomes [44]. More than half of these have been reduced to solitary LTRs, but some 280 elements have retained genetic sequences, most of which have been rendered defective through the acquisition of stop codons, frameshift mutations and deletions. The only known completely intact and functional HERV-W *env* locus appears to be the established ERVWE1, located at 7q21.2, which codes for the syncytin-1 complete envelope protein important to placentation.

Rolland had already reported that the MSRV surface unit *env *sequence, AF331500, was significantly different from that of the *env* of ERVWE1, showing 87% sequence homology [38]. Messenger RNA can be transcribed *in vitro *using reverse transcriptase to produce its complementary DNA sequence, which is known as cDNA. In the genomes of peripheral blood mononuclear cells (PBMCs) of MS patients and controls, Laufer *et al., *showed that almost 30% of the HERV-W *env *cDNAs extracted by this technique were the result of recombinations of HERV-W *env *elements from different chromosomal integrations sites. In the authors’ opinion these recombinations were most likely to have been generated *in vitro*. But viral recombination, a quintessentially symbiogenetic pattern of evolution, is also known to occur *in vivo*, for example in the origins of pandemic influenza, and, as indicated above, there have been suggestions of such recombination involving retroviruses in rodents and humans [1, 20, 21].

By mapping HERV-W *env *cDNA sequences from PBMCs of MS patients and healthy controls onto individual genomic HERV-W *env *elements, they identified seven transcribed HERV-W *env* loci in these cells. They confirmed that one of these was the HERV-W *env* locus, ERVWE1, coding for the placental syncytin-1, which, as already described by Antony, is transcribed in the mononuclear cells of both normal controls and patients with MS. They also showed that the sequence, AF123882, taken by Antony as representative of MSRV *env*, probably originates from a HERV-W *env* locus on chromosome 15 (15q21.3), and represents an incomplete or interrupted open reading frame. They further showed that the other five transcribed HERV-W loci also had various deletions and truncations of their genetic domains and LTRs. But among these they identified a second transcriptionally active HERV-W *env* element, located on the X chromosome (Xq22.3), which contained an almost complete *env* gene, its full ORF interrupted by just a single stop codon at amino acid codon position 39. The translatable *env *sequence from this locus would give rise to a truncated syncytin-like protein of 475 amino acids. The sequence, AF331500, regarded as the standard MSRV *env *surface unit, could be explained as a recombination of Xq22.3 and another defective HERV-W *env*, on chromosome 5. Putting it another way, the amino acid sequence of a recombinant MSRV *env* SU protein, the same ENV-SU shown by Rolland to have proinflammatory properties, and which was generated using the AF331500 MSRV *env *clone, appeared to be identical to the amino acid sequence of the HERV-W *env* protein that would be encoded by the Xq22.3 HERV-W *env *gene*. *

There was an additional inference. The stop codons in the two *env* sequences, AF331500 and Xq22.3, differed in a single nucleotide. The elimination of the stop codon at position 39 of the HERV-W *env *Xq22.3 would result in an uninterrupted full-length HERV-W *env *open reading frame capable of encoding a complete *env *protein that contained a signal peptide. This stop codon of HERV-W *env *Xq22.3 might readily be unstopped by recombination with other HERV-W *env* elements, which were shown to contain the necessary triplet at the right place. Laufer *et al., *also showed that the monoclonal antibody, 6A2B2, which had been shown to detect a HERV-W *env* antigen present in MS lesions, was now seen to be generated against a fragment of the Xq22.3 HERV-W *env* sequence. These findings raised the possibility that the antigen detected in the plaques of brains affected by MS could be encoded by the Xq22.3 HERV-W *env* locus.

## NEUROPHARMACOLOGICAL EXTRAPOLATIONS

It seems likely that one or more HERV-Ws, and in particular their *env* genes, are playing a significant role in the pathogenesis of MS. This does not necessarily imply that they are aetiological, but it does suggest that they are involved in a complex inflammatory interplay with other as yet not fully understood factors, including genetic predisposition, perhaps extrinsic factors such as stress, or exogenous viruses, and cytokines such as TNFα. The HERV research has reached the stage where it appears to offer potential new lines of neuro-pharmacological research. A single putatively complete virus, the MSRV, and three *env* genes , related to MSRV, the HERV-W discovered by Laufer *et al.,* on chromosome X (Xq22.3), and the HERV-W, also known as the ERVWE1 locus, found on chromosome 7 (7q21.2), are prime candidates for such study. If Laufer *et al., *are correct in their conclusions, the *env* of MSRV and the X located HERV-W may prove to be intrinsically the same, which might, after confirmation by further research, reduce the candidate *env* sequences to two. Moreover, if Mameli *et al.,* are supported in their most recent findings, the *env* sequence of the MSRV has a key 12 nucleotide insert in its trans-membrane moiety that might enable its discrimination from the *env *of the ERVWE1 virus in future research. The potential pharmacological extrapolations fall into two broad categories, diagnostic/ predictive and therapeutic. The following is intended just as a general guide and does not aim to be exhaustive.

### Diagnostic/ Predictive Extrapolations

Although the therapeutic options in MS have improved in recent years, currently there is no simple haematological diagnostic or prognostic assay that might assist clinicians in managing MS. Haematological assays of the MSRV has been associated with drug therapy outcomes and predictions of severity of disease and prognosis. Its reconstructed genomic organisation has recently been outlined by Mameli *et al.,* [43] and a highly specific *env* gene trans-membrane sequence has been reported. Both the putative virus and its *env *gene sequences have been repeatedly extracted from the plasma, supernatant of cultured PBMCs and CSF of patients suffering from MS. In the recent Mameli paper, albeit in small numbers of patients, relative quantification of the levels of MSRV *env *sequences showed marked differences between untreated MS patients and controls. In addition, approximately 70% of patients with optic neuritis, a disease that can be prodromic to MS, were reported by the same authors to be MSRV-positive in blood and CSF [45], although a previous study appears to have come to the very opposite conclusions [46]. Previous studies by the Italian group have also shown a dramatic reduction in MSRV RNA load and response to treatment with beta-interferon. They have also shown statistically significant correlations between the MSRV RNA load in plasma, PBMCs and CSF in relation to the clinical stage of MS and, in particular, progression of disease, with the CSF data further extrapolated to six years of follow-up [47]. This would suggest that there is potential for preliminary testing of *in vitro* diagnostic and predictive assays, for blood and for CSF, that should include MSRV, the predicted complete ORF of Xq22.3 *env* and the known *env *of ERVWE1, with the aim, initially, of confirming the usefulness of the assays and then, if confirmed, leading to standardised research and clinical laboratory assays.

### Therapeutic Extrapolations

The development of the same *in vitro* tests of blood and CSF might also extrapolate to response to treatment assays. There is some evidence that titres of MSRV in particular might help in predicting patients liable to more severe disease and a more aggressive long term prognosis [34]. This, in turn, might thus prove useful at a time when multiple therapies are available. A range of different pathogenetic mechanisms, involving both innate and adaptive immunity, have been shown to underlie relapse and progression of MS, and it is possible that different therapies may work in different ways to mitigate these mechanisms. At the same time the secondary-progressive pattern of disease has proved to be more aggressive and resistant to therapies, making new approaches urgent. In this situation an *in vitro* test system that helped to differentiate possible responders to different therapies at the onset of diagnosis and treatment would be helpful. This might, for example, assist in predicting those patients who will prove relatively refractory to single therapy, so that alternative or multiple therapy might be considered. Inoculation of peripheral blood lymphocytes (PBLs) of healthy controls with MSRV virion particles led to a significant polyclonal activation of T cells following the pathogenetic pattern of a superantigen [31]. The hybrid animal model experiments [32], with their suggestion of T-cell mediated immunopathogenicity [33], merit further extrapolation as does the work on the pro-inflammatory properties of the AF331500 ENV-SU clone taken as representative of MSRV by Rolland *et al.,* [38]. Such probing of innate and adaptive immune responses to MSRV *in vitro* could be extended to the three *env* sequences and include probing and correlating the effects of various known, and potentially new, therapeutic agents. The putative link between outcome and MSRV in CSF on presentation [33, 34, 47], if confirmed, could be extended, for example in patients suffering a more aggressive course of disease, where levels before and after different therapies, perhaps including multiple therapies, might prove helpful in predicting outcomes. Another line of investigation that might be worth further extrapolation is the evidence for a positive feedback loop affecting MSRV expression, and viral release from peripheral blood mononuclear cells stimulated by cytokines [33].

The work by Johnston *et al.,* linking cytokines, such as TNFα, to raised levels of HERVs might also prove useful to pharmacologists looking for means of *in vitro* experiment [39]. The elegant *in vitro* work by Antony *et al.,* [36] introducing an *env*-containing vector to cell cultures of foetal astrocytes, macrophages and oligodendrocytes, would appear also to offer a potential tool for assessment of the neuro-inflammatory properties of the MSRV and the three *env *sequences, and might augment the existing armamentarium for testing existing and novel therapeutic compounds.

The cooperative study of ERVWE1 regulation in astrocyte cell cultures, highlighting the role of cytokines, and inhibitory effect of beta-interferon, might also be worth extrapolating in the same way to existing and novel therapeutic compounds [40]. In particular it would be interesting to look in more detail at the subunit, p65, within the enhancer domain of the promoter, including the epigenetic work with siRNA, noting that the authors drew attention to this as a possible therapeutic/ pharmacological target. Pharmacologists interested in this arena might also note the marked similarity of cellular location, expression and potential neuro-toxicity of both MSRV and ERVWE1 in MS patients in different studies, which might suggest that both HERV-W members are significantly activated in the cerebral lesions of MS patients. But they should also bear in mind the current difficulty in differentiating between the two closely related *env* sequences and expressed products within cells, so the possibility remains that the unwonted expression of MSRV (?Xq22.3) *env* might dysregulate a physiological function of ERVWE1 syncytin-1.

## Figures and Tables

**Fig. (4):**
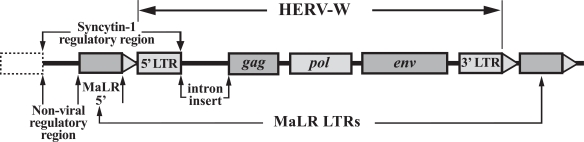
This genetic structure of the ERVWE1 locus contains a complete HERV-W, with its three genes *gag, pol *and *env,* together with the flanking LTRs. Whereas the *gag* and *pol* genes have been silenced by mutations, the *env* gene has been conserved by selection. The **env gene promoter is contained within the HERV-W 5’LTR, with the relevant genetic sequence also conserved. There is an intron insert between the *gag* and 5’LTR. The upstream regulation of the env gene is complex, including the MaLR 5’LTR and the non-viral regulatory region delineated in the figure. (After Gimenez J. and Mallet F. [19])
